# Comparing undesirable behaviours between ‘designer’ Poodle-cross dogs and their purebred progenitor breeds

**DOI:** 10.1371/journal.pone.0342847

**Published:** 2026-03-19

**Authors:** Gina T. Bryson, Dan G. O'Neill, Zoe Belshaw, Claire L. Brand, Rowena M. A. Packer

**Affiliations:** 1 Department of Clinical Science and Services, Royal Veterinary College, Hatfield, Hertfordshire, United Kingdom; 2 Department of Pathobiology and Population Sciences, The Royal Veterinary College, Hatfield, Hertfordshire, United Kingdom; 3 EviVet Evidence-based Veterinary Consultancy, Nottingham, United Kingdom; Universidad de Costa Rica, COSTA RICA

## Abstract

Designer-crossbreed dogs (deliberate cross-breeding between two or more pure breeds) are exploding in popularity, often driven by beliefs that they commonly exhibit desirable behaviours such as being easy to train or being good with children, despite minimal supporting evidence. This study aimed to fill this gap by comparing Canine Behavioural Assessment and Research Questionnaire (C-BARQ) scores between the three most common designer-crossbreeds in the UK and their relevant progenitor breeds. C-BARQ behaviour data for all 12 sub-scales were collected in March 2023 via an online questionnaire of owners of Cockapoo, Labradoodle, Cavapoo, Cocker Spaniel, Labrador Retriever, Cavalier King Charles Spaniel and Poodle dogs acquired aged ≤16 weeks from 1^st^ January 2019. C-BARQ scores were analysed using multivariable linear modelling. Valid responses were received representing 9,402 dogs. From 72 behavioural comparisons overall (3 designer-crossbreeds x 2 progenitors x 12 C-BARQ scales), designer-crossbreeds overall exhibited more undesirable behaviours than a progenitor breed in 44.4% comparisons and fewer undesirable behaviours in 9.7% comparisons, with no differences detected for the remaining 45.8%. Cockapoos displayed the most undesirable behaviours of the three designer crossbreeds, and differed from Cocker Spaniels and Poodles in 16/24 comparisons, scoring worse for all 16. Cavapoos differed from Cavalier King Charles Spaniels and Poodles in 12/24 comparisons, scoring worse in 11. In contrast, Labradoodles differed from Labrador Retrievers and Poodles in 11/24 comparisons, scoring worse than their progenitors in five behaviours, but better in six. These findings suggest notable behavioural differences between designer-crossbreeds versus their progenitor breeds, with Cockapoos and Cavapoos in particular scoring worse. Wider awareness by prospective owners of these potential issues around undesirable behavioural traits could avoid misbelief-driven acquisitions (e.g., designer-crossbreeds require minimal training, or are particularly suited to households with children) that risk public health (e.g., elevated dog bite risks) and relinquishment due to unmet expectations.

## Introduction

Over the past decade, the UK canine population has experienced sizeable demographic shifts towards an emerging dog type known as ‘designer-crossbreeds’, i.e., first or later generations of deliberate cross-breeding between two or more existing pure breeds to create new ‘hybrid’ breeds. Many of the most canine popular designer-crossbreeds are Poodle crosses, with the Cockapoo (Cocker Spaniel x Poodle) already in the top three most popular puppy breeds (pure or designer-crossbreeds) to own in the UK in 2019 [1], and then rising further in demand in the UK during the COVID-19 pandemic [2]. Previous research has identified factors including perceived enhanced health, ease to train and hypoallergenicity as motivators towards acquiring designer-crossbreed dogs rather than purebred dogs [1].

However, despite rapidly rising public demand for certain designer-crossbreed dogs, there is currently very little published research reporting their behaviour, even though breed-associated behaviours (actual or perceived) are a key factor influencing breed choice during pre-purchase decision-making [3,4]. Consequently, during pre-purchase research, prospective owners considering whether to acquire a specific designer-crossbreed are forced to rely on anecdotal information. Domestic dogs are reported to show considerable diversity in behaviour both within and between breeds [5]. Owners often seek out a breed they believe will suit their lifestyle and personalities, and behaviour-related perceptions are identified as a strong motivator of designer-crossbreed acquisition [1,6]. Owners of UK designer-crossbreed dogs (not just limited to Poodle-crosses) were more likely to seek a breed that they perceived as easy to train and/or suitable with children, compared to purebred breed owners [1]. Similarly, a US study of just Poodle-crosses reported that the assumption that a breed of dog was ‘good with children’ or a ‘good companion breed’ more highly influenced Poodle-cross owners when making their breed-choice compared to non-Poodle-cross owners (purebred and mixed breed owners), and owners of their purebred progenitor breeds specifically [6].

A key factor for sustained dog ownership and owner satisfaction is a tight match between owners’ expectations of their future breed/crossbreed’s behaviour prior to acquisition and the reality of ownership following acquisition. When prior expectations are partially or fully unmet, the dog-owner bond can be significantly impaired and make for a more challenging ownership experience, and even potential relinquishment or euthanasia [7]. Specifically, beliefs around certain breeds being predictably ‘good’ or ‘safe’ with children puts public health at risk, e.g., via increased paediatric bite risk when this expectation is not met, or when owners consequently fail to engage in appropriate safeguarding of children around dogs [8] or in appropriate dog training. In mitigation, better evidence on the scale of undesirable behaviours in designer-crossbreeds is urgently needed to support appropriate evidence-based acquisition and behavioural husbandry by owners, to promote good dog-owner relationships, to support dog welfare (e.g., by meeting their behavioural needs), and to maximise safety and wellbeing of all human members of households with dogs. Throughout this paper, the use of “undesirable behaviour” will refer to any canine behaviour that the owners of a dog perceive as problematic or troublesome [9].

Early work exploring behavioural comparisons between non-designer-crossbreeds (i.e., general crossbreed dogs) compared to purebred dogs suggested these non-specific crossbreeds were more likely to display undesirable behaviours compared to purebred breeds [10,11]. However, to date there have been minimal behavioural comparisons between designer-crossbreeds and their progenitor breeds. Current understanding of behavioural genetics would suggest that expression of particular behavioural traits in designer-crossbreeds would fall intermediately between the level of expression of each progenitor breed [12]. However, there is little published research supporting such a ‘regression to the mean’ theory in designer-crossbreeds. A recent study investigating undesirable behaviour expression in US designer-crossbreeds compared Goldendoodles and Labradoodles with their corresponding progenitor breeds: Golden Retriever, Labrador Retriever and either the Standard or Miniature Poodle [13]. That study used the Canine Behavioural Assessment and Research Questionnaire (C-BARQ), a validated and standardised behavioural tool, to compare fourteen different behavioural features [14]. Goldendoodles exhibited significantly more undesirable behaviours (i.e., higher C-BARQ scores for subscales where higher = less desirable behaviour) than the Poodle for two of the 14 C-BARQ traits: dog-directed aggression and dog-directed fear, and significantly more undesirable behaviour compared to Golden Retrievers for one trait, stranger-directed fear. In contrast, the Labradoodle exhibited significantly less undesirable behaviour than the Poodle for one trait, dog rivalry [14]. It is possible that these differences reflect a combination of effects that include the varying genetics of designer crossbreeds compared to their progenitor breeds (‘nature’) and may also reflect differences in dog ownership profiles and style between designer crossbred and purebred dog owners (‘nurture’), or underlying differences in their interpretation and scoring of behaviour. In the ten other C-BARQ behaviour categories, Labradoodles scored intermediately between their progenitor breeds, somewhat supporting regression to the mean for designer-crossbreed expression of undesirable behavioural traits to levels midway between the progenitor breeds. The Goldendoodle was also reported to express most behaviours studied at an intermediate level between the purebred progenitors, but the Goldendoodle varied from its progenitors more often compared to the Labradoodle. Consequently, these results suggest that behavioural profiles differ between distinct Poodle-crosses despite sharing half of the same parentage, and even when their non-Poodle progenitor is a similar breed (i.e., both progenitors were Retriever types in that study). This suggests that each distinct designer-crossbreed should be considered as a unique breed, and consequently require generation of their own unique evidence base. Further research utilising the C-BARQ to compare behaviour between designer-crossbreeds and their progenitors is needed to add evidence on the behavioural status of other popular Poodle-cross designer-crossbreeds, particularly in light of the rapidly rising popularity of Cockapoos and Cavapoos in the UK.

There is limited evidence regarding the inheritance of the two behavioural traits commonly perceived as desirable attributes of designer-crossbreeds, i.e., ease of training and suitability with children. The limited evidence that can be derived from studies of animals bred as working guide dogs, who are commonly intentional crosses between distinct pure breeds, is mixed. Training results from The Seeing Eye Guide Dog School (US) reported that Labrador Retriever x Golden Retriever cross dogs (also known as Goldadors) had higher probability for successfully completing training and graduating as guide dogs compared to their purebred progenitors [15]. In contrast, a questionnaire-based study assessing international guide dog puppy training identified Labrador Retriever x Golden Retriever cross dogs to have reduced success rate in training compared to other purebred breeds [16]. Those authors proposed that crossbreeding may result in a greater diversity of behaviour and therefore poorer success rate for outcomes that require consistent behavioural patterns across dogs.

Companion animal-related commercial organisations often cite a good temperament of many Poodle-crosses as a reason for recommending these dogs for homes with children [17–19], and in a previous questionnaire-based study, 41.6% of designer-crossbreeds were reported to live in households with children, compared to just 31.2% of purebreds [1]. However, no data on actual interactions between designer-crossbreeds and children have been published to date to support claims of higher suitability of designer-crossbreed behaviour for family homes compared to other breeds. Additional research is required to investigate the prevalence and characteristics of undesirable behaviours in designer-crossbreeds, and particularly behaviours that could pose a risk to children, e.g., human-directed aggression.

Given the rising ownership of certain designer-crossbreeds despite a very limited evidence base on their behaviours, the current study aimed to explore UK owner-reported undesirable behaviours in the three most popular Poodle-cross designer-crossbreeds (Cockapoos, Labradoodles and Cavapoos) and compare this to their relevant purebred progenitor breeds. The study objectives were to quantify and compare the expression of undesirable behaviours reported by UK owners in dogs aged up to five years between three designer-crossbreeds (Cockapoo, Labradoodle and Cavapoo) and each of their two relevant progenitor breeds (CKCS, Cocker Spaniel, Labrador Retriever and Miniature Poodle, Standard Poodle or Toy Poodle) using a validated, standardised canine behaviour questionnaire (C-BARQ). The study hypothesised that Cockapoo, Labradoodle and Cavapoo score intermediately in behavioural expression on all C-BARQ scales between their relevant progenitor breeds.

## Materials and methods

An online survey was used to explore ownership experiences for three selected designer-crossbreeds (Cockapoo, Labradoodle and Cavapoo) and their relevant progenitor purebred breeds (CKCS, Cocker Spaniel, Labrador Retriever and Miniature Poodle, Standard Poodle or Toy Poodle). The questionnaire included questions related to the dog’s health (reported elsewhere [20]), husbandry and behaviour, but only the behavioural data are analysed and reported here. The questionnaire was hosted using Research Electronic Data Capture (REDCap) software [21] and was open from 21^st^ February 2023–21^st^ April 2023. Respondents typically took 20−25 minutes to complete the questionnaire. The study received ethical approval from the Social Science Research Ethical Review Board at the Royal Veterinary College (URN SR2022−0184). Respondents gave written consent prior to starting the questionnaire. Respondents could exit the survey at any time, but because the survey was anonymous, respondents could not withdraw their response once submitted.

### Survey recruitment

Respondents were recruited by multiple pathways [1]. Digital posters for individual designer-crossbreeds and purebred progenitor breeds (S1 File) were created and shared by the authors via snowball sampling on breed-specific pages on social media sites (e.g., Facebook, Instagram, Reddit and X). Large UK animal charities, namely Blue Cross, RSPCA, and PDSA, veterinary-specific organisations (e.g., VetPartners Ltd), and other organisations from the veterinary and welfare sectors (e.g., PetPlan, Pets at Home, Agria and APDAWG) promoted the questionnaire on their social media platforms. The pet classifieds site, Pets4Homes, shared the questionnaire link by direct email to users who had shown an interest in purchasing any of the study breeds/crossbreeds in the 600 days prior to the survey launch. The UK Kennel Club promoted the questionnaire via their registered breeders for the six purebred breeds. Finally, the authors distributed breed-specific flyers in-person at Crufts 2023, Birmingham. A complete list of disseminators is shown in the Acknowledgements section.

### Inclusion criteria

Respondents were required to be aged 18 years or over, resident in the UK and to currently own at least one designer-crossbreed from a specified list of Poodle crosses [Cockapoo (Cocker Spaniel crossed with a Poodle), Cavapoo (CKCS crossed with a Poodle), Labradoodle (Labrador Retriever crossed with a Poodle)] or a purebred dog of a progenitor breed of the aforementioned designer-crossbreeds [CKCS, Cocker Spaniel, Labrador Retriever, Miniature Poodle, Standard Poodle or Toy Poodle]. The three designer-crossbreeds were chosen as the most numerous designer-crossbreeds in VetCompass in the UK, to maximise the relevance and statistical power of the study results [22].

Study dogs were required to have been acquired aged 16 weeks or younger from 1^st^ January 2019 onwards. This ensured all study dogs were aged under 5 years old at completion of the questionnaire to reduce age confounding effects on health [23,24] and behaviour (e.g., avoiding geriatric-onset behavioural problems [25]), given that the wider designer-crossbreed population is likely to be younger on average than their purebred counterparts owing to the recent popularity spike in designer-crossbreed dogs. As undesirable behaviours are most commonly reported in dogs under three years old [26], this age range aimed to still capture the expression of many undesirable behaviours in the general population. Respondents with more than one eligible dog were asked to complete the questionnaire for their dog named first alphabetically.

### Survey content

The sections of the questionnaire relevant to the current study included:

1. The first section captured owner demographics, including age, gender, postcode and occupation, and canine demographics, including breed, sex, date of birth and their generation (if the dog was a crossbreed).
2. To collect data on dog behaviour, the Canine Behavioural Assessment and Research Questionnaire (C-BARQ) [14] was used to explore six broad aspects of dog behaviour: training and obedience, aggression, fear and anxiety, separation-related behaviour, excitability, and attachment and attention-seeking. C-BARQ is a well-established tool that includes 73 questions which are aggregated during analysis into 12 numerical subscales within the C-BARQ^(100)^ scoring method [5]. To limit the length of the survey and to reduce response drop-out, questions under the ‘miscellaneous problem behaviours’ category of the original C-BARQ survey were omitted. These items have previously been shown to be optional and removable from the questionnaire without compromising the reliability or validity of the remaining subscales [1].
3. To quantify which (if any) resources were used by the owner to help with dog behaviour and training, and whether the owner had ever sought professional advice for their dog’s behaviour, further questions were either adapted from previous surveys published by the current research group [2,27,28], or were newly curated by the same team. These questions were presented as single-choice or multi-choice with an additional option for free-text responses.
4. To explore between owners’ expectations of behaviour and training prior to acquisition compared to the reality after acquisition of their dog, two additional multi-choice questions were included with an option for free-text responses to expand upon their answers.

The full study questionnaire also included questions regarding the dog’s health and husbandry that are not reported here. The full questionnaire can be found in S2 File.

### Data analysis

The questionnaire data were manually cleaned in Microsoft Excel (2013). Respondents were excluded if their responses did not show that they met all of the inclusion criteria. Statistical analysis used IBM SPSS Statistics (V29.0.0.0) software. Responses were combined to represent all three Poodle-type breeds (Toy, Miniature and Standard) as one Poodle group for analysis purposes, to maximise statistical power for comparisons [1]. Descriptive statistics reported frequency and percentage for relevant variables. Median, range and interquartile range (IQR) of dog age (years) were calculated at the date of completion of the questionnaire. Other demographic variables were compared between designer-crossbreeds and their progenitors using chi-square testing.

Analysis of variance (ANOVA) was used to compare mean breed scores for each of the 12 C-BARQ subscales. The two-way ANOVA models included a fixed set of additional variables to account for confounding that were selected using an information theory approach [29] developed from other canine welfare studies. The following were included as confounding factors [30,31]: dog age, sex and neuter status, owner gender and age, whether the puppy had been seen with the mother on day of collection (as a proxy of illegal sales [27]), whether the owner worked in the canine sector (given potentially differing experience levels/perceptions of behaviour), whether the respondents was the primary dog owner, first-time ownership and health disorder prevalence (given the emerging relationship between health and behaviour [32] using VetCompass coding [33]. The *p* value for statistical significance for all analyses was set at *p* < 0.05.

## Results

From *n* = 10,524 responses received, *n* = 1,113 (10.6%) did not meet all the inclusion criteria and *n* = 9 (0.1%) had missing data for some of the inclusion criteria, leaving 9,402 (89.3%) valid responses that met all the inclusion criteria and were included in the final analytic dataset.

### Dog demographics

The *n* = 9,402 dogs in the final analysis included *n* = 3,424 (36.4%) designer-crossbreed and *n* = 5,978 (63.6%) purebred dogs. The designer-crossbreed dog group comprised of *n* = 1,856 (19.7% of 9,402 dogs) Cockapoos, *n* = 583 (6.2%) Labradoodles and *n* = 985 (10.5%) Cavapoos. The purebred dog group comprised of *n* = 715 (7.6%) CKCS, *n* = 2,237 (23.8%) Cocker Spaniels, *n* = 2,099 (22.3%) Labrador Retrievers, *n* = 352 (3.7%) Miniature Poodles, *n* = 315 (3.4%) Standard Poodles and *n* = 260 (2.8%) Toy Poodles, with the three Poodle breeds combined represented by *n* = 927 dogs (9.9%).

All dogs included in the study were required to be 5 years old or under. The overall median age of all dogs in the study was 22 months (IQR: 12–34, range: 9–56). Designer-crossbreeds were significantly younger than purebred breeds (*p* < 0.001), with the median age of dogs within the designer-crossbreed group being 19 months (IQR: 10–31, range: 1–55), while the median age of dogs in the purebred breed group was 24 months (IQR:13–32, range: 1–56).

Of the designer-crossbreeds with data available, *n* = 1,899 (20.2%) were reported as F1 generation (first-generation cross between two purebred progenitor breeds), *n* = 225 (2.4%) F2 generation, *n* = 32 (0.3%) F3 generation and *n* = 143 (1.5%) multigenerational. The full details of designer-crossbreed generation can be found in Table 1. An F1 (*n* = 1,899, 20.2%) was the most commonly reported generational type overall across all three designer-crossbreeds. Labradoodles were significantly less likely to be reported as F1 compared to the other two designer-crossbreeds (F1: Cockapoo: *n* = 1,103, 59.7%; Cavapoo: *n* = 600, 61.3%; Labradoodle: *n* = 196, 33.8%; *p* < 0.001). A total of *n* = 154 (1.6%) owners reported they did not know their dog’s generation, and the generation of *n* = 365 (3.9%) designer-crossbreeds was unspecified.

**Table 1:** Frequency of generation types for Cockapoo (*n* = 1,847), Labradoodle (*n* = 580) and Cavapoo (*n* = 978) designer-crossbreeds in the UK.

Generation of Cross	Overall for three Designer-crossbreeds No. (%)	Cockapoo No. (%)	Labradoodle No. (%)	Cavapoo No. (%)
**F1** (e.g., their parents were a purebred Poodle (Toy/Miniature/Standard) and a purebred second breed (e.g., a Cavalier King Charles Spaniel, Cocker Spaniel, Labrador Retriever))	1,899 (55.5%)	1103 (59.7%)	196 (33.8%)	600 (61.3%)
**F1B** (e.g., Their parents were an F1 cross and a purebred Poodle (Toy/Miniature/Labrador))	605 (17.7%)	312 (16.9%)	103 (17.8%)	190 (19.4%)
**F1BB** (e.g., Their parents were an F1B cross and a purebred Poodle (Toy/Miniature/Standard))	43 (1.3%)	18 (1.0%)	16 (2.8%)	9 (0.9%)
**F2** (e.g., Their parents were both F1 crosses)	225 (6.6%)	156 (8.4%)	38 (6.6%)	31 (3.2%)
**F2B** (e.g., Their parents were an F2 cross and a purebred Poodle (Toy/ Miniature/Standard))	69 (2.0%)	43 (2.3%)	18 (3.1%)	8 (0.8%)
**F2BB** (e.g., Their parents were an F2B cross and a purebred Poodle (Toy/Miniature/Standard))	11 (0.3%)	3 (0.2%)	7 (1.2%)	1 (0.1%)
**F3** (e.g., Their parents were both F2 crosses)	32 (0.9%)	6 (0.3%)	21 (3.6%)	5 (0.5%)
**Multigeneration** (e.g., Their parents were both a cross of any generation beyond F3)	143 (4.2%)	7 (0.4%)	131 (22.6%)	5 (0.5%)
**Unknown**	378 (11.0%)	199 (10.8%)	50 (8.6%)	129 (13.2%)

Of the *n* = 3,424 Poodle-cross designer-crossbreeds, *n* = 3,059 had information on their dog’s specific type of Poodle progenitor breed. Of these, Miniature Poodles (*n* = 1,749, 57.2%) were the most represented progenitor Poodle breed. Compared to the other two designer-crossbreeds, Cockapoos were most likely to include a Miniature Poodle progenitor (Cockapoo: *n* = 1,031, 55.8%; Cavapoo: *n* = 467, 47.7%; Labradoodle: *n* = 251, 44.1%; *p* < 0.001). Toy Poodles (884, 28.9%) were the second most represented progenitor Poodle type in the sample. Of the three designer-crossbreed types, Cavapoos were the most likely to include a Toy Poodle progenitor (Cavapoo: *n* = 406, 41.5%; Cockapoo: *n* = 442, 23.9%; Labradoodle: *n* = 36, 6.3%; *p* < 0.001). Standard Poodles (*n* = 426, 13.9%) were the least commonly represented Poodle progenitor in the sample. Of the three designer-crossbreed types, Labradoodles were the most likely to include a Standard Poodle progenitor (Labradoodle: *n* = 228, 40.1%; Cockapoo: *n* = 163, 8.8%; Cavapoo: *n* = 35, 3.6%; *p* < 0.001).

The overall study population included *n* = 4,373 (46.5%) male and *n* = 4,287 (45.6%) female dogs; the sex of *n* = 742 (7.9%) dogs was unspecified. Designer-crossbreeds were significantly more likely to be female than purebred dogs (designer-crossbreed: female *n* = 1,638, 51.3%, male: *n* = 1,558, 48.7%; purebred: female: *n* = 2,648, 48.5%, male: *n* = 2,815, 51.5%; *p* = 0.013). Sex distribution did not statistically differ significantly between the three designer-crossbreeds (male: Cockapoo *n* = 870, 50.1%, Cavapoo *n* = 429, 46.9%, Labradoodle *n* = 259, 47.4%; *p* = 0.237). Canine demographic data for each dog breed and total frequencies can be found in S3 File.

The overall study population included *n* = 4,785 (50.9%) entire dogs and *n* = 3,869 (41.1%) neutered dogs, while the neuter status of *n* = 748 (8.0%) dogs was unspecified. Designer-crossbreeds were more likely to be neutered than purebreds (designer-crossbreeds: *n* = 1,650 (51.6%), purebreds: *n* = 2,219 (40.6%); *p* < 0.001). Neuter status did not significantly differ between the three designer-crossbreeds (Cockapoo *n* = 909, 52.4%, Cavapoo *n* = 460, 50.2%, Labradoodle *n* = 281, 51.6%; *p* = 0.558).

Overall, *n* = 7,432 (79.0%) of the study population were insured, and *n* = 1,238 (13.2%) were uninsured. The insurance status of *n* = 732 (7.8%) dogs was unspecified. Designer-crossbreeds were more likely to be insured than purebreds (designer-crossbreeds *n* = 2,826 (82.5%), purebreds *n* = 4,606 (77.1%); *p* < 0.001). The probability of being insured did not statistically differ between the three designer-crossbreeds (insured: Cockapoo *n* = 1,535, 88.4%, Cavapoo *n* = 803, 87.6%, Labradoodle *n* = 489, 89.6%; *p* = 0.516).

In total, *n* = 6,697 (71.2%) of the study population saw the puppy’s mother when they went to pick their puppy up, and *n* = 1,282 (13.6%) did not see the puppy’s mother, with the remaining *n* = 1,423 (15.2%) unspecified. Designer-crossbreeds owners were significantly less likely to see the puppy with its mother when they collected their puppy than purebred owners (designer-crossbreeds *n* = 2,415 (82.7%), purebreds *n* = 4,282 (84.6%); *p* < 0.027). The probability of seeing the puppy’s mother at collection did not statistically differ between the owners of the three designer-crossbreeds (Cockapoo owners *n* = 1,319, 83.1%, Cavapoo *n* = 684, 83.1%, Labradoodle *n* = 412, 80.9%; *p* = 0.501).

The overall median health disorder prevalence of all dogs in the study was two disorders (IQR: 1–3, range: 0–13). The median disorder prevalence did not significantly statistically differ between designer crossbreeds and their purebred progenitors (designer crossbreeds: IQR: 1–3, range: 0–13, median: 2.0; purebreds: IQR: 1–3, range: 0–12, median: 2.0, *p* = 0.294), nor did it significantly statistically differ between the three designer-crossbreeds (Cockapoo: IQR: 1–3, range, 0–11, median: 2.0; Cavapoo: IQR: 0–3, range: 0–9, median: 2.0; Labradoodle: IQR: 1–3, range: 0–13, median: 2.0; *p =* 0.501).

### Owner demographics

From *n* = 10,524 responses received, *n* = 1,113 (10.6%) did not meet all the inclusion criteria and *n* = 9 (0.1%) had missing data for some of the inclusion criteria, leaving 9,402 (89.3%) valid responses that met all the inclusion criteria in the final analytic dataset.

Most respondents were female (*n* = 7,493, 79.7%), with *n* = 1,012 male (10.8%), *n* = 16 ‘other’ (0.2%) and *n* = 28 (0.3%) selecting ‘prefer not to say’. Compared to purebred dogs, designer-crossbreed owners were more likely to be female than male (designer-crossbreed female owners: *n* = 2,798, 88.7% vs. purebred female owners: *n* = 4,693, 87.0%, *p* = 0.020). The most represented age group among all respondents was 45–54 years old (23.5%), with no significant difference in owner age group between designer-crossbreed owners and purebred owners (for example, over 35 years: designer-crossbreed owners: *n* = 2,345, 76.0%; purebred owners: *n* = 4,019, 75.7%, *p* = 0.562).

Overall, 60.5% (*n* = 5,132) of respondents were first-time dog owners, while the remaining 39.5% (*n* = 3,353) had previously owned a dog. Designer-crossbreed dogs were significantly more likely to belong to first-time dog owners compared to purebred dogs (designer-crossbreeds: *n* = 1,543, 49.6% vs. purebreds: *n* = 1,803, 33.7%, *p* < 0.001). Adult-only homes were the most common household structure (*n* = 4,864, 51.7%), although designer-crossbreeds were significantly more likely to live in households with children than purebred dogs (living with children: designer-crossbreeds: 36.6% vs. purebreds: 29.4%; *p* < 0.001). Almost 1 in 10 respondents (*n* = 810; 9.5%) worked in the canine/animal care sector, with owners of designer-crossbreed dogs significantly less likely to work in this sector compared to purebred owners (designer-crossbreeds: *n* = 153, 4.9% vs purebreds: *n* = 657, 12.1%; *p* < 0.001). Owner demographic data for each dog breed and total frequencies can be found in S4 File.

#### C-BARQ behavioural comparisons.

A total of 72 C-BARQ behaviour scale comparisons were carried out: each of the 12 C-BARQ subscales were compared for each of the three designer-crossbreeds against their two relevant progenitor breeds (12 x 3 x 2 = 72). The full C-BARQ results for each breed can be found in the S5 File. After accounting for confounding in multivariable analyses, designer-crossbreeds overall differed significantly from a progenitor breed in 54.2% (*n* = 39/72) of behaviour scale score comparisons, with 41.7% (*n* = 30/72) showing higher and 12.5% (*n* = 9/72) showing lower behaviour scale scores. With the exception of trainability, higher scores equate to more undesirable behaviour. In contrast, higher trainability equates to less undesirable behaviour (i.e., higher trainability scores being taken as being more desirable), as such, results are reported as more or less undesirable behaviour to aid consistency of interpretation. Taking this into account, from 72 behavioural comparisons overall (3 designer-crossbreeds x 2 progenitors x 12 C-BARQ scales), designer-crossbreeds overall exhibited more undesirable behaviours than a progenitor breed in 44.4% (32/72) comparisons and fewer undesirable behaviours in 9.7% (7/32) comparisons, with no differences detected for the remaining 45.8% (33/72).

#### Cockapoo vs. progenitor breeds.

**Fig 1:**
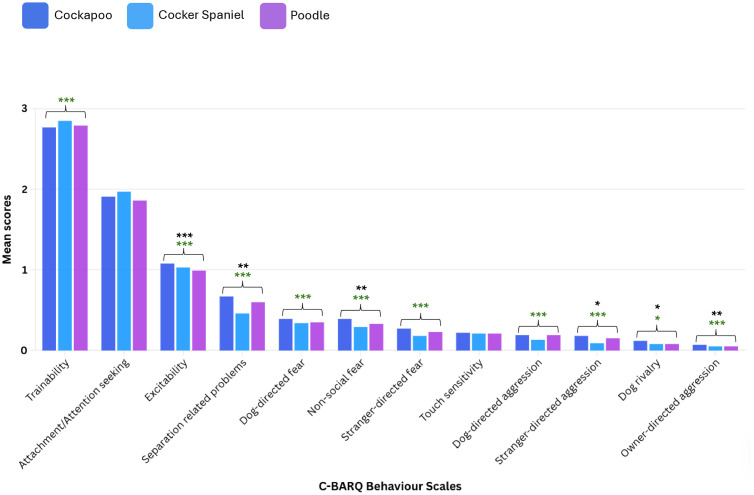
The mean scores for each of the 12 C-BARQ behaviour scales for Cockapoos, Cocker Spaniels and Poodles. Significance is shown by stars: *p = 0.05-0.01, **p = 0.01-0.001, ***p < 0.001. Stars in green indicate a significant difference between the designer-crossbreed and non-Poodle progenitor behaviour comparison. Stars in black indicate a significant difference between the designer-crossbreed and Poodle progenitor behaviour comparison.

Compared to their Poodle progenitor breed, Cockapoos differed in 6/12 (50.0%) of C-BARQ behaviour scales (Fig 1). Cockapoos scored significantly higher (i.e., exhibited more undesirable behaviour) in all six of these C-BARQ comparisons to Poodles: owner-directed aggression (*p* = 0.005), stranger-directed aggression (*p* = 0.017), dog rivalry (*p* = 0.024), non-social fear (*p* = 0.001), separation-related problems (*p* = 0.010) and excitability (*p* < 0.001).

Compared to their Cocker Spaniel progenitor breed, Cockapoos differed in 10/12 (83.3%) of C-BARQ behaviour scales after accounting for confounding in multivariable analyses. Cockapoos exhibited significantly more undesirable behaviour for 10/10 differing C-BARQ comparisons to Cocker Spaniels: owner-directed aggression (*p* < 0.001), stranger-directed aggression (*p* < 0.001), dog-directed aggression (*p* < 0.001), dog rivalry (*p* = 0.032), stranger-directed fear (*p* < 0.001), non-social fear (*p* < 0.001), dog-directed fear (*p* < 0.001), separation-related problems (*p* < 0.001), excitability (*p* < 0.001) and trainability (*p* < 0.001). No significant difference was found between Cockapoos compared to either progenitor breed (Poodles and Cocker Spaniels) for two C-BARQ behaviour scales: touch sensitivity (Cockapoo vs. Poodle: *p* = 0.457; Cockapoo vs. Cocker Spaniel: *p* = 0.959) and attachment/attention-seeking (Cockapoo vs. Poodle: *p* = 0.635; Cockapoo vs. Cocker Spaniel: *p* = 0.418).

#### Labradoodle vs. progenitor breeds.

**Fig 2:**
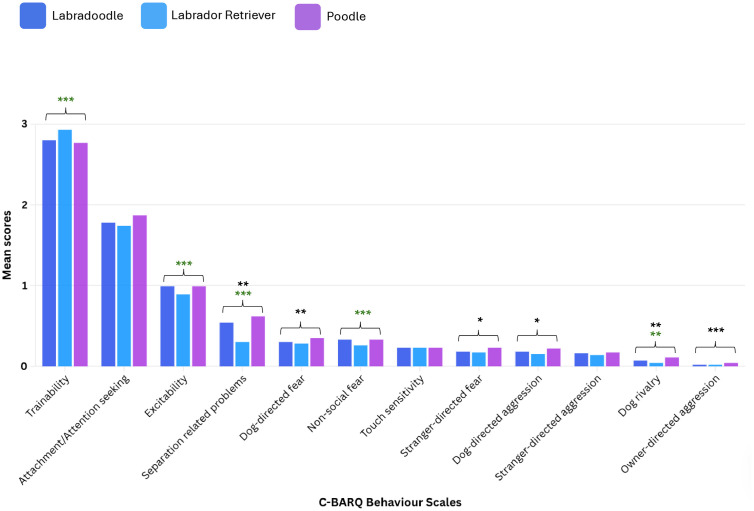
The mean scores for each of the 12 C-BARQ behaviour scales for Labradoodles, Labrador Retrievers and Poodles. Significance is shown by stars: *p = 0.05-0.01, **p = 0.01-0.001, ***p < 0.001. Stars in green indicate a significant difference between the designer-crossbreed and non-Poodle progenitor behaviour comparison. Stars in black indicate a significant difference between the designer-crossbreed and Poodle progenitor behaviour comparison.

Compared to their Poodle progenitor breed, Labradoodles differed in 6/12 (50.0%) C-BARQ behaviour scales after accounting for confounding in multivariable analyses (Fig 2). Labradoodles exhibited significantly less undesirable behaviour in 6/6 of these C-BARQ behaviour scale comparisons to Poodles: owner-directed aggression (*p* < 0.001), dog-directed aggression (*p* = 0.025), dog rivalry (*p* = 0.007), stranger-directed fear (*p* = 0.032), dog-directed fear (*p* = 0.009) and separation-related problems (*p* = 0.008).

Compared to their Labrador Retriever progenitor breed, Labradoodles differed in 5/12 (41.7%) C-BARQ behaviour scales after accounting for confounding in multivariable analyses. Labradoodles exhibited significantly more undesirable behaviour for 5/5 differing C-BARQ scale comparisons to Labrador Retrievers: dog rivalry (*p* = 0.009), non-social fear (*p* < 0.001), separation related problems (*p* < 0.001), excitability (*p* < 0.001) and trainability (*p* < 0.001). No significant difference was found between Labradoodles compared to either progenitor breed (Poodles and Labrador Retriever) for three C-BARQ behaviour scales: stranger-directed aggression (Labradoodle vs. Poodle: *p* = 0.364; Labradoodle vs Labrador Retriever: *p* = 0.086), touch sensitivity (Labradoodle vs. Poodle: *p* = 0.983; Labradoodle vs. Labrador Retriever: *p* = 0.652) and attachment/attention-seeking (Labradoodle vs. Poodle: *p* = 0.220; Labradoodle vs. Labrador Retriever: *p* = 0.456).

#### Cavapoo vs. progenitor breeds.

**Fig 3:**
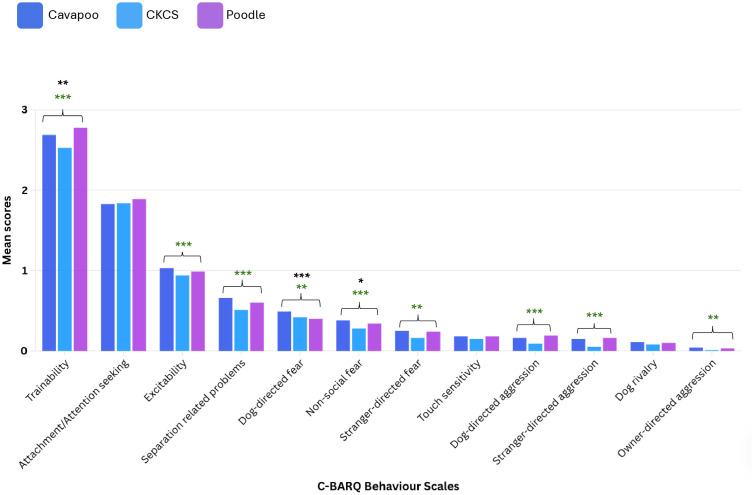
The mean scores for each of the 12 C-BARQ behaviour scales for Cavapoos, Cavalier King Charles Spaniels and Poodles. Significance is shown by stars: *p = 0.05-0.01, **p = 0.01-0.001, ***p < 0.001. Stars in green indicate a significant difference between the designer-crossbreed and non-Poodle progenitor behaviour comparison. Stars in black indicate a significant difference between the designer-crossbreed and Poodle progenitor behaviour comparison.

Compared to their Poodle progenitor breed, Cavapoos differed in 3/12 (25.0%) C-BARQ behaviour scales after accounting for confounding in multivariable analyses (Fig 3).

Cavapoos exhibited significantly more undesirable behaviour for 3/3 differing C-BARQ comparisons to Poodles: non-social fear (*p* = 0.006), dog-directed fear (*p* = 0.027) and trainability (*p* = 0.006).

Compared to their CKCS progenitor breed, Cavapoos differed in 9/12 (75.0%) C-BARQ behaviour scales after accounting for confounding in multivariable analyses. Cavapoos exhibited significantly more undesirable behaviours for 8/9 of the differing C-BARQ comparisons to CKCS: owner-directed aggression (*p* = 0.003), stranger-directed aggression (*p* < 0.001), dog-directed aggression (*p* < 0.001), stranger-directed fear (*p* = 0.002), non-social fear (*p* = 0.002), dog-directed fear (*p* = 0.003), separation-related problems (*p* < 0.001) and excitability (*p* < 0.001). Cavapoos exhibited significantly less undesirable behaviour for 1/9 of the differing C-BARQ comparisons to CKCS: trainability (*p* < 0.001). There was no significant difference between the Cavapoo compared to either progenitor breed (CKCS and Poodle) for C-BARQ scale scores for three behaviours: dog rivalry (Cavapoo vs. Poodle: *p* = 0.455; Cavapoo vs. CKCS: *p* = 0.085), touch sensitivity (Cavapoo vs. Poodle: *p* = 0.969; Cavapoo vs. CKCS: *p* = 0.118) and attachment/attention-seeking (Cavapoo vs. Poodle: *p* = 0.325; Cavapoo vs. CKCS: *p* = 0.860).

**Behavioural professional advice:** Overall, 17.2% (*n* = 1,072) of owners had sought advice from a behavioural professional regarding their dog’s behaviour. Cockapoo owners (*n* = 243, 22.7%) did not statistically differ from Cocker Spaniel owners (*n* = 219, 20.4%,95% CI: 18.0–22.8) or Poodle owners (*n* = 105, 9.8%, 95% CI: 8.0–11.6, *p* = 0.137) in whether they had sought advice from a behavioural professional regarding their dog’s behaviour. Labradoodle owners (*n* = 92, 8.6%) had higher odds for whether they had sought advice from a behavioural professional regarding their dog’s behaviour than Poodle owners (*n* = 105, 9.8%, OR:1.44, 95% CI: 8.0–11.6, *p* = 0.022) but did not differ from Labrador Retrievers (*n* = 250, 23.3%, 95% CI: 20.8–25.8). Cavapoo owners (*n* = 104, 9.7%) did not statistically differ from CKCS owners (*n* = 59, 5.5%, 95% CI: 4.2–6.8) or Poodle owners (*n* = 105, 9.8%, 95% CI: 8.0–11.6, *p* = 0.939) in whether they had sought advice from a behavioural professional regarding their dog’s behaviour.

**Training methods:** Overall, *n* = 1,126 (11.9%) owners used exclusively “rewards-only” methods of training, compared to *n* = 5,088 (53.8%) of owners that used both “reward and aversive” methods.

Compared to Poodle owners (rewards-only: *n* = 151, 13.4%; reward and aversive: *n* = 454, 8.9%), there was no significant difference in the methods of training used by Cockapoo owners (rewards-only: *n* = 279, 24.8%; reward and aversive, *n* = 941, 18.5%, *p* = 0.648). However, Cocker Spaniel owners (rewards-only: *n* = 200, 17.8%; reward and aversive: *n* = 1286, 25.3%) had higher odds of using “reward and aversive” based training methods than just “rewards-only” methods, compared to Cockapoo owners (OR: 1.98, 95% CI: 1.61–2.45, *p* < 0.001). Compared to Poodle owners, there was no significant difference in the methods of training used by Labradoodle owners (rewards-only: *n* = 76, 6.7%; rewards and aversive: *n* = 319, 6.3%, *p* = 0.127); however, Labrador Retriever owners (rewards-only: *n* = 169, 15.0%; reward and aversive: *n* = 1,282, 25.2%) had higher odds of using “reward and aversive” based training methods than “rewards-only” methods, compared to Labradoodle owners (OR: 1.87, 95% CI: 1.37–2.56, *p* < 0.001). Cavapoo owners (reward-only: 133, 11.8%; reward and aversive: *n* = 475, 9.3%) did not differ from either Poodle owners (*p* = 0.267) or CKCS owners (reward-only: 118, 10.5%; reward and aversive: *n* = 328, 6.4%, *p* = 0.165) in their use of training methods (Table 2).

**Table 2:** Multivariable logistic regression analysis results comparing the probability of designer-crossbreed owners using both reward and aversive-based training methods compared to the progenitor purebred owners. *Multivariable modelling also included age of dog, sex of dog, age of owner, owner gender, whether puppy had been seen with the mother on day of pick up, whether owner worked in the canine sector, primary dog ownership, and first-time ownership. Figures in bolds indicate p < 0.05.

Designer-Crossbreed	Progenitor Purebred	Multivariable*		
		Odds Ratio	95% CI	P value
Cockapoo	Poodle	0.95	0.74–1.20	*p* = 0.648
	Cocker Spaniel	1.98	1.61–2.45	***p* < 0.001**
Labradoodle	Poodle	0.78	0.56–1.07	*p* = 0.127
	Labrador Retriever	1.87	1.37–2.56	***p <* 0.001**
Cavapoo	Poodle	0.85	0.64–1.13	*p* = 0.267
	Cavalier King Charles Spaniel	0.81	0.60–1.09	*p* = 0.165

**Fig 4:**
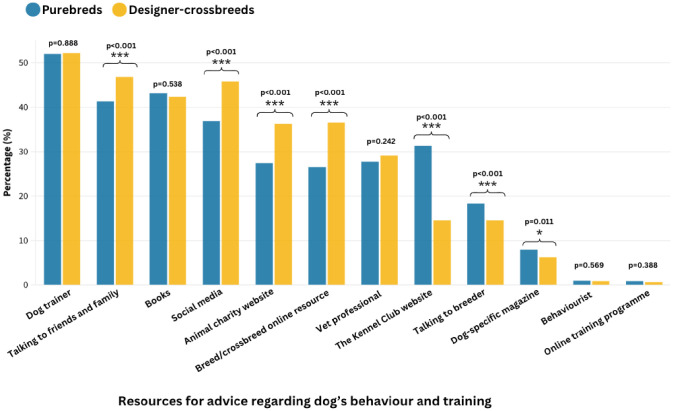
The percentage of designer-crossbreed owners compared to purebred owners that have ever used specific resource(s) for advice regarding their dog’s behaviour and training.

**General Training Advice/Resources:** The most commonly used training advice/resources used overall were dog trainers (*n* = 3,206, 33.9%), talking to friends and family (*n* = 2,662, 28.2%), books (*n* = 2,635, 27.9%), social media (*n* = 2,466, 26.1%) and animal charity websites (*n* = 1,881, 19.9%) (Fig 4).

Of the training advice and resources that significantly differed between designer-crossbreeds and their purebred progenitor breeds (Table 3), all purebred owners had higher odds of using The Kennel Club website as a training and behaviour resource, compared to designer-crossbreed owners (Poodle vs Cockapoo: OR: 3.09, 95% CI: 2.42–3.93, *p* < 0.001; Cocker Spaniel vs Cockapoo: OR: 2.99, 95% CI: 2.45–3.65, *p* < 0.001; Poodle vs Labradoodle: OR: 2.41, 95% CI: 1.75–3.31, *p* < 0.001; Labrador Retriever vs Labradoodle: OR: 2.33, 95% CI: 1.93–3.45, *p* < 0.001; Poodle vs Cavapoo: OR: 2.33, 95% CI: 1.77–3.08, *p* < 0.001, CKCS vs Cavapoo: OR: 1.92, 95% CI: 1.42–2.60, *p* < 0.001).

**Table 3:** Multivariable logistic regression analysis results comparing the probability of designer-crossbreed owners using the seven most common types of training and behaviour resource/advice compared to the progenitor breed owners. Multivariable modelling also included age of dog, sex, age of owner, owner gender, whether puppy had been seen with the mother on day of pick up, whether owner worked in the canine sector, primary dog ownership, and first-time ownership. Red coloured cells denote purebred owners having higher odds of using that training resource, whereas blue coloured cells denote designer-crossbreed owners having higher odds at using that specific training resource. Figures in bolds indicate a statistically significant result. * OR odds ratio **CI confidence interval.

Training and behaviour resource/advice	Purebred progenitors vs Cockapoo						Purebred progenitors vs Labradoodle						Purebred progenitors vs Cavapoo					
	Poodle			Cocker Spaniel			Poodle			Labrador Retriever			Poodle			CKCS		
	OR*	Mean 95% CI**	P value	OR*	Mean 95% CI**	P value	OR*	Mean 95% CI**	P value	OR*	Mean 95% CI**	P value	OR*	Mean 95% CI**	P value	OR*	Mean 95% CI**	P value
The Kennel Club website	3.09	2.42-3.93	***p* < 0.001**	2.99	2.45-3.65	***p* < 0.001**	2.41	1.75-3.31	***p* < 0.001**	2.58	1.93-3.45	***p* < 0.001**	2.33	1.77-3.08	***p* < 0.001**	1.92	1.42-2.60	***p* < 0.001**
An animal charity website	0.62	0.50-0.78	***p* < 0.001**	0.73	0.62-0.86	***p* < 0.001**	0.50	0.38-0.65	***p* < 0.001**	0.56	0.45-0.71	***p* < 0.001**	0.60	0.46-0.76	***p* < 0.001**	0.76	0.58-0.99	***p* = 0.043**
A breed/ crossbreed-specific online resource	0.60	0.48-0.75	***p* < 0.001**	0.72	0.61-0.85	***p* < 0.001**	0.51	0.39-0.67	***p* < 0.001**	0.53	0.42-0.66	***p* < 0.001**	0.55	0.43-0.70	***p* < 0.001**	0.65	0.50-0.85	***p* = 0.002**
Social media sites	0.75	0.61-0.91	***p* = 0.005**	0.81	0.69-0.94	***p* = 0.007**	0.83	0.64-1.08	*p* = 0.166	0.67	0.53-0.84	***p* < 0.001**	0.66	0.52-0.83	***p* < 0.001**	0.62	0.48-0.80	***p* < 0.001**
Dog-specific magazine	1.09	0.75-1.58	*p* = 0.874	1.02	0.76-1.38	*p* = 0.667	1.23	0.74-2.03	*p* = 0.068	1.51	0.97-2.35	*p* = 0.430	1.72	1.06-2.82	***p* = 0.030**	1.47	0.85-2.52	*p* = 0.169
Talking to friends or family who own or had owned a dog	0.65	0.53-0.80	***p* < 0.001**	0.87	0.75-1.01	*p* = 0.067	0.73	0.57-0.95	***p* = 0.019**	0.89	0.71-1.11	*p* = 0.306	0.71	0.57-0.90	***p* = 0.004**	0.63	0.49-0.81	***p* < 0.001**
Talking to a dog breeder	1.97	1.53-2.53	***p* < 0.001**	1.28	1.03-1.58	***p* < 0.001**	1.40	1.02-1.92	***p* = 0.038**	1.04	0.78-1.39	*p* = 0.769	1.83	1.36-2.46	***p* < 0.001**	1.02	0.72-1.44	*p* = 0.930

Poodle owners had higher odds of using dog-specific magazines as a training and behaviour resource compared to Cavapoos (OR: 1.72, 95% CI: 1.06–2.82, *p* = 0.030), but no significant difference was found between the other designer-crossbreeds and their progenitor purebred breeds (CKCS vs Cavapoo: *p* < 0.169; Poodle vs Cockapoo: *p* = 0.874; Cocker Spaniel vs Cockapoo: *p* = 0.667; Poodle vs Labradoodle: *p* = 0.068; Labrador Retriever vs Labradoodle: *p* = 0.430).

Both Poodle and Cocker Spaniel owners had higher odds of using dog breeders as a training and behaviour resource, compared to Cockapoo owners (Poodle vs Cockapoo: OR:1.97, 95% CI: 1.53–2.53, *p* < 0.001; Cocker Spaniel vs Cockapoo: OR:1.28, 95% CI: 1.03–1.58, *p* < 0.001). Poodle owners had higher odds of using dog breeders as a training and behaviour resource compared to Labradoodle owners, but no significant difference was found between Labrador owners and Labradoodle owners (Poodle vs Labradoodle: OR: 1.40, 95% CI: 1.02–1.92, *p* = 0.038; Labrador Retriever vs Labradoodle: *p* = 0.769). Poodle owners had higher odds of using dog breeders as a training and behaviour resource compared to Cavapoo owners (Poodle vs Cavapoo: OR: 1.83, 95% CI: 1.36–2.46, *p* < 0.001), but no significant difference was found between CKCS owners and Cavapoo owners (CKCS vs Cavapoo: *p* = 0.930)

In contrast, all designer-crossbreed owners had higher odds of using an animal charity website as a training and behaviour resource compared to purebred owners (Poodle vs Cockapoo: OR:0.62, 95% CI: 0.50–0.78, *p* < 0.001; Cocker Spaniel vs Cockapoo: OR:0.73, 95% CI: 0.62–0.86, *p* < 0.001; Poodle vs Labradoodle: OR: 0.50, 95% CI: 0.38–0.65, *p* < 0.001; Labrador Retriever vs Labradoodle: OR: 0.56, 95% CI: 0.45–0.71, *p* < 0.001; Poodle vs Cavapoo: OR: 0.60, 95% CI: 0.46–0.76, *p* < 0.001; CKCS vs Cavapoo: OR: 0.76, 95% CI: 0.58–0.99, *p* = 0.043).

Additionally, all designer-crossbreed owners had higher odds of using a breed/crossbreed specific online resource, as a training and behaviour resource, compared to purebred owners (Poodle vs Cockapoo: OR:0.60, 95% CI: 0.48–0.75, *p* < 0.001; Cocker Spaniel vs Cockapoo: OR:0.72, 95% CI: 0.61–0.85, *p* < 0.001; Poodle vs Labradoodle: OR: 0.51, 95% CI: 0.39–0.67, *p* < 0.001; Labrador Retriever vs Labradoodle: OR: 0.53, 95% CI: 0.42–0.66, *p* < 0.001; Poodle vs Cavapoo: OR: 0.55, 95% CI: 0.43–0.70, *p* < 0.001; CKCS vs Cavapoo: OR: 0.65, 95% CI: 0.50–0.85, *p* = 0.002).

Cockapoo owners had higher odds of using social media sites as a training and behaviour resource compared to their progenitor breed owners (Poodle vs Cockapoo: OR:0.75, 95% CI: 0.61–0.91, *p* = 0.005; Cocker Spaniel vs Cockapoo: OR:0.81, 95% CI: 0.69–0.94, *p* = 0.007). Labradoodle owners had higher odds of using social media sites as a training and behaviour resource compared to Labrador Retrievers (Labrador Retriever vs Labradoodle: OR: 0.67, 95% CI: 0.53–0.84, *p* < 0.001) but no significant difference was found between Labradoodle and Poodle owners (*p* = 0.166). Cavapoo owners had higher odds of using social media sites, as a training and behaviour resource compared to their progenitor breed owners (Poodle vs Cavapoo: OR: 0.66, 95% CI: 0.52–0.83. *p* < 0.001; CKCS vs Cavapoo: OR: 0.62, 95% CI: 0.48–0.80, *p* < 0.001).

Cockapoo owners had higher odds of talking to friends or family who own or had owned a dog as a training and behaviour resource compared to Poodle owners (Poodle vs Cockapoo: OR: 0.65, 95% CI: 0.53–0.80, *p* < 0.001) but no significant difference was found between Cockapoo owners and Cocker Spaniel owners (*p* = 0.067). Labradoodle owners had higher odds of talking to friends or family who own or had owned a dog as a training and behaviour resource compared to Poodle owners (Poodle vs Labradoodle: OR: 0.73, 95% CI: 0.57–0.95, *p* = 0.019) but no significant difference was found between Labradoodle owners and Labrador Retriever owners (*p* = 0.306). Cavapoo owners had higher odds of talking to friends or family who own or had owned a dog as a training and behaviour resource compared to their progenitor breeds (Poodle vs Cavapoo: OR: 0.71, 95% CI: 0.57–0.90, *p* = 0.004; CKCS vs Cavapoo: OR: 0.63, 95% CI: 0.49–0.81, *p* < 0.001).

## Discussion

This study identified several differences in the levels of undesirable behaviours in three specific designer-crossbreed breeds (Cockapoo, Labradoodle and Cavapoo) compared to their purebred progenitor breeds. Previous analyses of the same population of study dogs identified relatively few differences in physical health between the same three designer-crossbreeds compared to their progenitor breeds [1]. However, in sharp contrast, the three designer-crossbreeds overall differed in 54.2% (39/72) of behaviour comparisons to their progenitor breeds. Of the 39 comparisons that differed, designer-crossbreeds exhibited more undesirable behaviours in 82.1% (32/39) of behaviour scales compared and exhibited less undesirable behaviours in just 17.9% (7/39) of behaviour scales, despite perceived positive behavioural traits being a major driver of acquisition for designer crossbreed dogs [1].

Several patterns were notable in the behaviours that differed between the designer Poodle-crosses and the purebred parents. All three designer-crossbreed breeds exhibited significantly more non-social fear (e.g., fear of inanimate stimuli such as traffic, loud/sudden noises, traffic, novel objects/contexts), separation-related problems (e.g., undesirable behaviours such as excessive vocalisation and destructiveness when separated from their owner) and excitability (e.g., strong reactions to exciting/arousing events such as walks, car trips, doorbells and visitors) compared to their non-Poodle progenitors. Fear-related behaviours represent potentially serious welfare concerns that could reflect poorer emotional health in these dogs and could negatively impact the dog-owner bond [34] and owner wellbeing [35]. However, several earlier studies [36] have shown that remarkably few owners seek veterinary or other professional help or advice to manage these three important behavioural issues. A UK-based study investigating fear responses to noises in domestic dogs revealed fewer than a third of dog owners sought professional advice regarding treating their dog’s response to noises [37]. Owners of designer-crossbreeds should be mindful that displaying these behaviours likely indicates their dog is experiencing negative emotions such as fear, anxiety and/or panic which should be prioritised with similar consideration as any physical ailment to maintain good welfare. Canine behaviourists and veterinary professionals can support designer-crossbreed owners by increasing awareness of the serious negative impacts of these undesirable behaviours upon their dogs and offer appropriate therapies to address them [38].

### Behavioural variability between individual designer-crossbreed breeds

There was considerable variability in the levels of undesirable behaviours shown between the three designer-crossbreed breeds. This suggests that designer-crossbred dogs should not be considered as a homogenous group from a behavioural perspective. Cockapoos displayed the most undesirable behaviours of the three designer-crossbreeds studied (compared to the purebred progenitors). When compared to Cocker Spaniels and Poodles (2 x 12 C-BARQ behaviours), Cockapoos differed in 16/24 behaviour scale comparisons, exhibiting more undesirable behaviour in all 16 comparisons. Cavapoos differed in 12/24 behavioural scale comparisons (2 x 12 C-BARQ behaviours), exhibiting more undesirable behaviour for 11/12 differing comparisons. In contrast, Labradoodles differed in 11/24 behaviour scale comparisons (2 x 12 C-BARQ behaviours) but only displayed more undesirable behaviour than their progenitors in five comparisons, while exhibiting less undesirable behaviour in six comparisons. With the exception of better trainability in Cavapoos compared to one of its progenitor breeds (the CKCS), the Labradoodle was the only designer-crossbreed to show less undesirable behaviour than its purebred parents in ≥ 1 behaviour scale. Consequently, from these results it is evident each new designer-crossbreed needs its own evidence base and extrapolating information from other designer-crossbreeds should be done with caution.

The results of the current study offer little support for the study hypothesis that specific Poodle-crosses score intermediately for all C-BARQ scales compared to their relevant progenitor breeds. That study hypothesis was based on an earlier C-BARQ analysis which reported that Labradoodles scored intermediately between their parent breeds, the Poodle and Labrador Retriever [13]. Instead, our results suggest that the behaviour of this emerging designer Poodle-crossbreed demographic is generally worse than their progenitor breeds. However, in the current study, it was evident that Labradoodles differed less in the undesirable behaviour comparisons with their purebred progenitors, unlike the other designer-crossbreeds. This somewhat supports the findings of Shouldice et al (2019) in Labradoodles and highlights the importance of acknowledging disparity between designer-crossbreed breeds rather than expecting all Poodle-crosses to exhibit the same behaviours.

### Factors influencing behavioural differences between designer crossbreeds and purebred progenitors

The reasons behind this disparity between Labradoodles and the other two smaller designer-crossbreeds are not clear, but one possibility is that Labradoodles were established earlier than Cockapoos and Cavapoos (as evidenced by the lowest level of F1 crosses in this study) and have undergone more generations of selection towards more desirable behavioural phenotypes than the other two designer crossbreeds. The impact of genetic inheritance of certain behaviours should be further considered. For example, studies exploring the presence of Insulin-like Growth Factor-1 (IGF1) in smaller sized dog breeds suggest that the IGF-1 gene may have pleiotropic effects in miniature and toy dog breeds, causing these dog types to display more fearful, excitable or reactive behaviours [10,39,40]. The Cockapoo and Cavapoo are physically smaller sized dogs owing to their Poodle progenitor being more likely to have been the Toy or Miniature Poodle (e.g., 41.50% of Cavapoos in the current study had Toy Poodle parentage), with serum levels of IGF-1 protein found to correlate with body size in Toy, Miniature and Standard Poodles [39]. This could partly explain why the Cockapoo and Cavapoo display more undesirable behaviours compared to the Labradoodle as a larger designer-crossbreed. Size is an influential factor in undesirable behaviour, with previous studies finding that small dogs were twice as likely to show human-directed aggression compared to large dogs, with 30.8% of small dogs exhibiting this behaviour compared to 14.5% of large dogs and 19.4% of medium dogs [40]. That same study found that small (23.1%) and medium (25.8%) size dogs more commonly exhibited aggression toward objects in movement compared with large dogs (10.8%). Other owner-reported studies have similarly reported small dogs to be rated as more disobedient, more excitable and more nervous by their owners [10,41]. A correlation between small dog size and higher levels of undesirable behaviours could also reflect environmental influences, given behaviour is a multifactorial trait and that owners are reported to interact differently with smaller versus larger dogs. For example, small dogs have been reported to receive less formal training than large dogs [41,42] and are walked less frequently [43]. In addition, owners of smaller dogs have been reported to be more inconsistent in interactions with their dog and engage less in training and play activities than owners of larger dogs [43]. Responses to owners may also differ based on dog size, with increased anxiety and fear found to be associated with more frequent use of punishment in smaller dogs but not in larger dogs [43].

Given that small size dog breeds have been growing in popularity in the past decade [44,45], with new Poodle-cross breeds such as Cavapoos a large contributor to this trend [2], it is possible that the prevalence of undesirable behaviours in the overall dog population could also increase. Results from the current study are particularly important when considering the varying roles in which smaller designer-crossbreed dog types are commonly used. In recent years, the use of designer-crossbreeds as therapy dogs has grown, especially with neurodivergent children who may intensively handle their dogs. Cavapoos make up 16.6% of dogs used by ‘Dogs for Autism’, a registered charity providing Autism Assistance dogs in the UK [46]. If, as this study’s results suggest, Cavapoos demonstrate higher levels of aggression behaviours than the CKCS, their appropriateness in such roles, as well their role as a companion animal with children in general, given their high representation in such homes [1], may need to be reconsidered.

### Cockapoo behavioural profile

Cockapoos were reported to display considerably more undesirable behaviours in comparison to their progenitor breeds in the current study than Cavapoos and Labradoodles. Cockapoos scored significantly higher than both purebred progenitors in aggression-related behaviours, including owner-directed aggression, stranger-directed aggression and dog rivalry. One explanation for this disparity could be a differential effect of the Cocker Spaniel parentage in the Cockapoo compared to the Cavalier King Charles Spaniel parentage in the Cavapoo. There have been numerous studies documenting the prevalence of aggressive behaviours in Cocker Spaniels (often termed “Cocker Rage”), particularly Golden Cocker Spaniels [47–49]. The combination of being a smaller breed and Cocker Spaniel parentage could explain why Cockapoos display an exacerbated level of aggression-related behaviours than their progenitors and other Poodle-mix designer-crossbreeds. Furthermore, although both Labrador Retrievers and Cocker Spaniels are from the gun dog group, the differing roles for which they were originally bred could impact their overall temperament. Labrador Retrievers, as the name suggests, were originally bred to retrieve game and therefore to work closely with their handler, staying still and alert until released. Anecdotally retrieving breeds have transitioned from field working roles to domestic companion roles more easily than spaniel breeds [50]; however, empirical research is needed to confirm this. Limited data suggests differences in impulsivity between show and working line Labrador Retrievers [51,52]. It is unknown whether any of the Labrador Retriever progenitors of the Labradoodles in this study, or the purebred Labrador Retrievers, were from working or show lines. Future research could explore differences in Labradoodles derived from each line to evaluate which are more suitable for companion homes and show lower levels of undesirable behaviours. In contrast, the Cocker Spaniel breed was developed to flush out game from dense areas of woodland and grassland, and therefore required high-energy levels, mental resilience and intelligence. The result is a breed that is anecdotally described as an “interesting mixture of brains, energy, speed and independence” which can be “more of a problem in pet homes” [53]. When bred with a Poodle, these behavioural tendencies, combined with the smaller resultant crossbred offspring, could potentially result in more undesirable behaviours.

Consolidating the current study’s findings regarding Cockapoo behaviour, the responses also revealed a higher percentage of Cockapoo owners sought professional behavioural advice compared to Labradoodle and Cavapoo owners. Indeed, Cockapoo owners were the second most likely of all six breeds involved in the study to seek professional advice for their dog’s behaviour (with Labrador owners being the most likely). This may imply that Cockapoos display a high level of undesirable behaviours which owners struggle to manage on their own and thus seek help to resolve. Until now, reports of undesirable behaviour in Cockapoos compared to other designer-crossbreeds has been predominantly anecdotal [54,55]. Undesirable behaviours are associated with a higher risk of relinquishment in dogs [56,57]. Spaniel Aid, a UK based charity focused on rescuing and rehoming of spaniels and spaniel-crosses, cite a lack of owner research as a main reason for relinquishment [58]. This risk of rehoming has the potential to increase if prospective owners continue to acquire breeds without informing and preparing themselves for likely behavioural traits. Spaniel and spaniel-crosses are often advertised as a *“very good choice for first-time owners”* and can be *“easily trained”* [59–61] but the current findings refute these claims*.* Any owner interested in purchasing a Cockapoo should therefore now be aware that this breed may be predisposed to exhibiting aggressive behaviours.

### Owner-reported behaviour

It is important to note that the behaviour data analysed in the current study was owner-reported. Human-centric factors have been found to influence each owner’s perception of their dog’s behaviour. An Australian study exploring owner interactions with companion dogs reported that dog owners believed crossbreds displayed more disobedient, nervous and excitable behaviours compared to purebreds [10]. To try to account for any such information biases, the multivariable statistical modelling used in the current study to analyse the C-BARQ data included several covariates to account for effects from human factors such as gender and ownership experience. For example, designer-crossbreeds were more likely to be owned by first-time owners compared to purebreds. First-time ownership has previously been reported as a risk factor for higher levels of owner-reported behavioural problems [10,42,62] and was also shown in the current study to have a significant association in numerous behaviour comparisons between designer-crossbreeds and purebreds. The current C-BARQ results revealed that first-time owners reported their dogs, regardless of breed type, had worse trainability as well as higher separation-related problems and higher non-social fear scores. Additionally, the findings reveal the owner’s age and whether the respondent worked in the canine/animal care sector also had significant associations with C-BARQ scores, with younger owners reporting higher levels of dog-directed aggression and higher separation-related problems, but also higher trainability scores. In contrast, respondents that worked in the canine/animal care sector reported lower touch sensitivity and lower social fear scores, consolidating previous research into canine behaviour that found an association with dogs displaying a lower level of undesirable behaviours and owners working in the canine sector [63]**.**

### Impact of health on behaviour

Overall disorder prevalence was the covariate with the greatest association within the ANOVA analysis of behaviour subscale scores. Overall disorder prevalence is a proxy summary metric for overall health which is increasingly recognised as an important contributing factor towards higher undesirable behaviour scores in dogs, particularly disorders associated with stress and pain [64–70]. Large-scale empirical studies have reported statistical associations between the presence of specific health problems and undesirable behaviours. For example, atopic dermatitis in dogs has been reported as associated with higher levels of undesirable behaviours such as excitability and reduced trainability [68], and anxiety and fear-driven behaviours [71]. Designer-crossbreeds in the current study cohort had higher odds of skin disorders such as atopic dermatitis and otitis externa than their progenitors [20] which may contribute to their elevated levels of undesirable behaviours. These associations between physical health problems and undesirable behaviours suggest that owners of designer-crossbreeds who perceived problematic behaviours in their dogs should first consider seeking veterinary advice to diagnose any underlying physical health conditions that could be contributing to their dog’s behaviours.

### Influence of provenance on behaviour

Another covariate that had notable associations with behaviour was whether the owner had seen the puppy with its mother on the day of collection, a proxy of illegal puppy sales in England under Lucy’s Law [72]. Not seeing the mother on collection of the puppy was linked to lower trainability scores in all comparisons between the designer-crossbreeds and their progenitor breeds. Additionally, not seeing the puppy’s mother on collection of the puppy was associated with higher scores of owner-directed aggression and excitability in the Cockapoo and progenitor breed comparisons, higher separation-related problem scores in the Labradoodle and progenitor breed comparisons and higher dog rivalry scores in the Cavapoo and progenitor breed comparisons. This reinforces previous results that indicate viewing a puppy without their mother is associated with a higher level of undesirable behaviour in the future [73]. The current study’s results further consolidate earlier findings [1] that designer-crossbreed owners are less likely to see the mother of the puppy on the day of collection compared to purebred owners. By neglecting this key pre-purchasing behaviour, designer-crossbreed owners not only risk supporting the illegal puppy trade, but also compromising their own dog’s welfare*.* Evidence suggests purchasing puppies from poor welfare sources has long-term negative behavioural and health outcomes, including for puppies purchased from pet shops [62,74,75] puppy farms [76], purchased without their mother and father present [73,77], or purchased underage [78]. All dog owners, especially designer-crossbreed owners, must therefore adhere to puppy purchasing guidance.

### Gun dog behaviour and training

Cocker Spaniels and Labrador Retrievers are classified as gun dogs, one of the seven breed groups recognised by the UK Kennel Club [79]. Owners of both Cocker Spaniel and Labrador Retriever dogs were significantly more likely to use a combination of both reward-and-aversive than rewards-only based training methods compared to the Poodle and associated designer-crossbreed owners. The original role of gun dogs to locate and/or retrieve live game necessitated high levels of training. A major early training “guideline” (1952) for gun dogs was a book by Moxon (1952) “Gundogs: Training and Field Trials” [80] that promoted methods that teach the dog “right” and “wrong” via the precise administration of physical punishment. While from the mid-20th century onwards, training of companion dogs more generally in the UK shifted to focus more on building relationships using reward-based methods, training cultures for gun dogs and working dogs have anecdotally remained focused on functionality and utility, perpetuating use of aversive methods. This is despite aversive training methods (using positive punishment and negative reinforcement) having since been criticised for harming dog welfare and safety in the wider sector, e.g., [81,82], supported by scientific studies documenting negative welfare affects [83–86].

Both Labrador Retrievers and Cocker Spaniels in the current study scored better in trainability than their designer Poodle-crossbreed counterparts; however, while seeming apparently a positive result, this effect may result from forced suppression of ‘disobedient’ behaviours that can increase stress and cause a more negative mood state [86,87]. Many owners use aversive training techniques for perceived faster and more effective outcomes than positive reinforcement training methods [83]. However, research shows reward-based training is not only more effective than aversive training, but it can also help build a stronger dog-owner relationship and improve trust [83,84,88]. Consequently, owners of Cockapoos and Labradoodles (crossbreeds with gun dog parentage) should not interpret the finding of increased trainability in these breeds to suggest that aversive methods will help with their dog’s training. Instead, owners are encouraged to seek professional and welfare-focused training guidance from either accredited dog behaviourists, or if inaccessible (e.g., due to finances) consult reliable online resources including animal welfare charities, e.g., Dogs Trust or Battersea Dogs and Cats Home, who provide free online training and behaviour resources that the public can readily access [89,90].

### Professional behavioural advice seeking

Designer-crossbreed owners had higher odds of using non-professional training and behaviour resources such as social media and information from family and friends than the owners of purebred dogs. These sources have the potential to be highly variable in quality and may be misleading [91]. Both designer-crossbreed owners and purebred owners were also found to use dog trainers as a common source for behaviour and training.

Dog training is currently unregulated in the UK and therefore there are no standards dog trainers have to adhere to [92], raising concerns over the training methods being promoted [93]. Dog owners are encouraged to seek professional advice regarding their dog’s behaviour, such as qualified behaviourists [94]. Furthermore, commonly accessed resources such as The Kennel Club should be encouraged to provide evidence-based information about the behaviour of designer-crossbreeds as well as purebred breeds, considering they are often only one or two generations away from the purebred breeds that they register.

### Limitations

The current study had some limitations despite the steps taken to mitigate as many as possible. Although owner-reported surveys can offer large and well-powered studies, dog owners generally have a poorer understanding of canine behaviour compared to professionals and therefore, behaviour data reported by owners may be less reliable than data reported by qualified behaviourists [7]. The use of a standardised and validated canine behaviour tool such as the C-BARQ helped to mitigate differences in owner-reported data regarding dog behavioural traits. The convenience sampling methods used in the current study could have promoted some selection bias because owners who were offered the option to participate may not fully represent all dog owners. It is also possible that owners who responded to the current survey may be more welfare-conscious than the wider population of dog owners in the UK, resulting in a ‘best case scenario’ representation for all breeds studied. Similarly, owners who had previously encountered problems or behavioural issues with their dogs may have been more motivated to complete the survey questionnaire about their dog’s behaviour and training. To minimise this selection bias, the current survey was promoted broadly across a range of social media platforms, including both breed-specific and non-breed-specific groups. It was also shared through diverse organisations with large followings and promoted in general pet or animal groups to reach a wider audience. In addition, the current paper focused on relative results between the breeds rather than on absolute values, with the assumption that respondent selection biases were likely to apply similarly across all the breeds.

The number of Poodles was relatively low in the study compared to the other purebred breeds. Consequently, the responses for each Poodle-type breed were combined and analysed as a collective rather than separately. However, the behaviour profiles of these three types of Poodles may differ, so this grouping process may have introduced bias between the results for the three designer-crossbreed breeds that were shown to differ in the different Poodle variant progenitors [5]. Additionally, it is important to note that the designer-crossbreeds involved in this study were Poodle-crosses, and consequently the results may not be applicable to other designer-crossbreeds that do not have Poodle parentage.

## Conclusions

The current study showed significant disparity for the behaviours exhibited between the three designer-crossbreeds studied and their purebred progenitors, with these three designer-crossbreeds overall showing more undesirable behaviours. In particular, Cockapoos exhibited significantly more undesirable behaviours than the Labradoodle and Cavapoo designer-crossbreeds. The results of this study highlight the importance of owners thoroughly exploring the characteristics of any breed or crossbreed during pre-purchase research to avoid mis-informed breed selection. In addition, prospective owners should meet the parents of any prospective puppy and appraise their behaviour before making any final acquisition decision. Veterinary professionals can use the findings of this study to support both prospective and current Poodle-cross designer-crossbreed owners by increasing awareness of undesirable behaviours in these specific designer-crossbreeds. Although this study provides a comprehensive and timely evaluation of the behaviour of the UK’s three most common designer-crossbreeds, given the growing popularity of designer crossbreeds more widely, and paucity of research on this topic, further studies are warranted to test and expand the results reported.

## Supporting information

S1 File Breed specific posters. (DOCX)

S2 File Designer-Crossbreed Questionnaire. (DOCX)

S3 File Canine demographic information. (DOCX)

S4 File Owner demographic information. (DOCX)

S5 File C-BARQ tables. (DOCX)
