# Supplementary figures and images for: Comparing undesirable behaviours between ‘designer’ Poodle-cross dogs and their purebred progenitor breeds
Source: PLoS One. 2026 Mar 19;21(3):e0342847. doi: 10.1371/journal.pone.0342847 (PMC13001074; doi:10.1371/journal.pone.0342847)

**S1 File. Breed specific posters.**


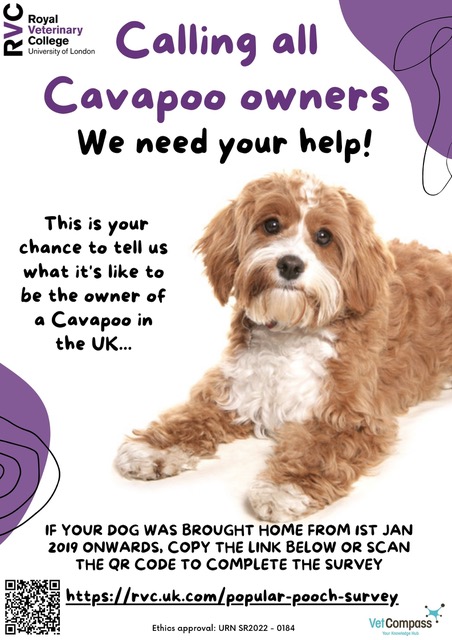


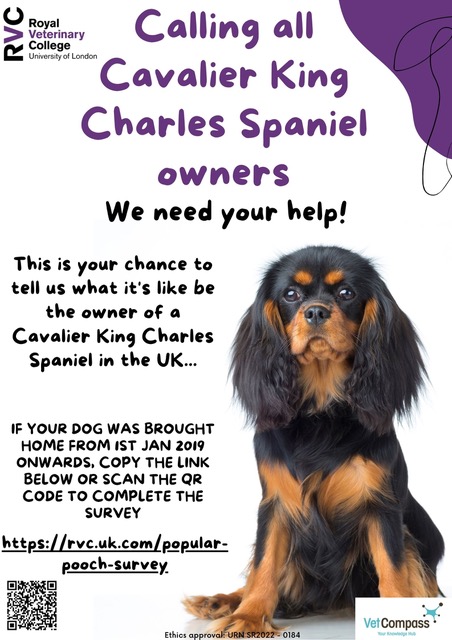


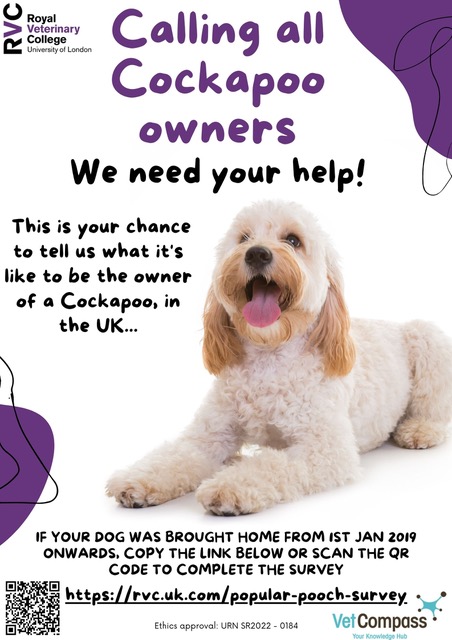


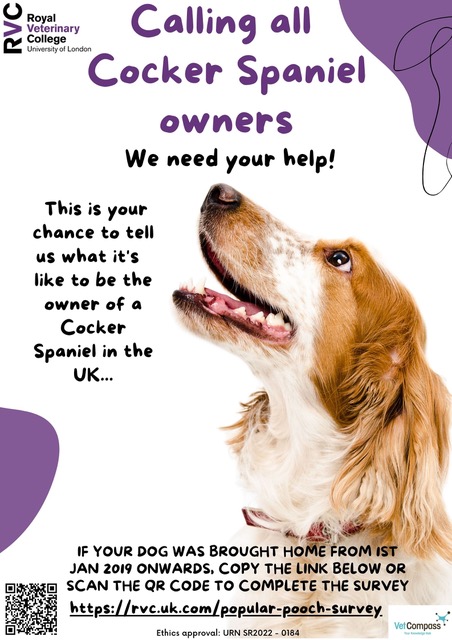


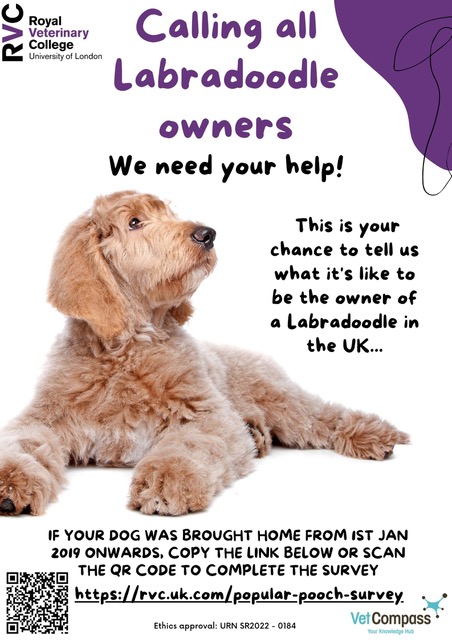


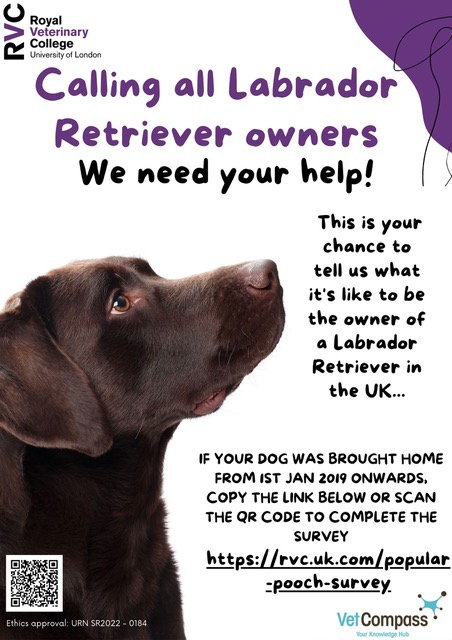


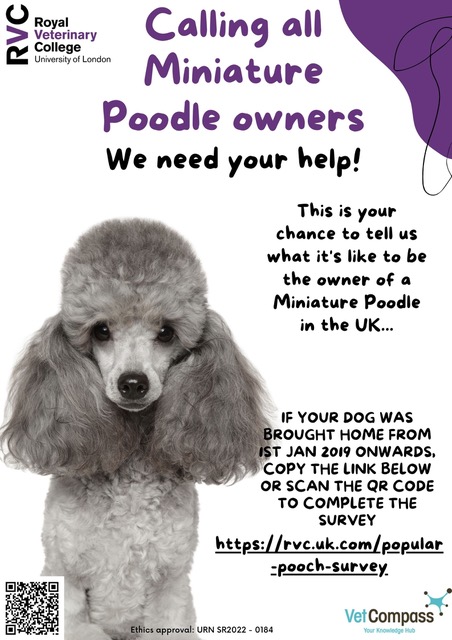


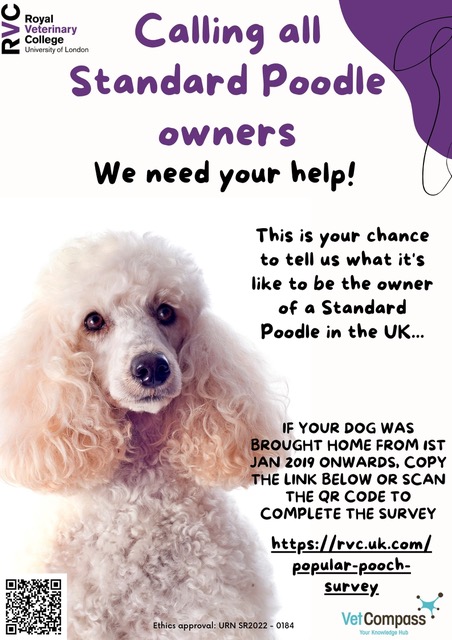


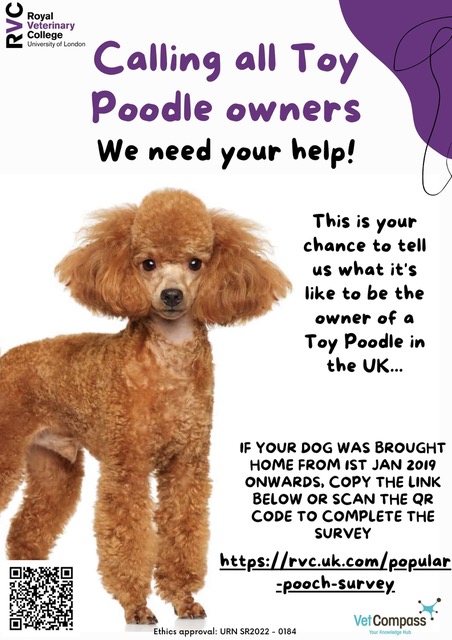

Supplement: S1 File — (DOCX) [file pone.0342847.s001.docx]
