# Supplementary material for: Comparing undesirable behaviours between ‘designer’ Poodle-cross dogs and their purebred progenitor breeds
Source: PLoS One. 2026 Mar 19;21(3):e0342847. doi: 10.1371/journal.pone.0342847 (PMC13001074; doi:10.1371/journal.pone.0342847)
Supplement: S3 File — (DOCX) [file pone.0342847.s003.docx]

| Dog breeds | Frequency | Sex | | Neuter status | | Insured status | |
| --- | --- | --- | --- | --- | --- | --- | --- |
|  |  | **Male** | **Female** | **Neutered** | **Not neutered** | **Insured** | **Not insured** |
| Cavapoo | 985 | 429 | 485 | 460 | 456 | 803 | 114 |
| CKCS | 715 | 354 | 283 | 319 | 319 | 535 | 103 |
| Cockapoo | 1856 | 870 | 866 | 909 | 825 | 1535 | 202 |
| Cocker Spaniel | 2237 | 1049 | 1002 | 737 | 1311 | 1770 | 284 |
| Labradoodle | 583 | 259 | 287 | 281 | 264 | 489 | 57 |
| Labrador Retriever | 2099 | 984 | 943 | 838 | 1089 | 1655 | 274 |
| Miniature Poodle | 352 | 163 | 169 | 123 | 208 | 262 | 70 |
| Standard Poodle | 315 | 154 | 135 | 104 | 185 | 214 | 76 |
| Toy Poodle | 260 | 111 | 116 | 98 | 128 | 169 | 58 |
| Total | 9402 | 4373 | 4286 | 3869 | 4785 | 7432 | 1238 |
