# Supplementary material for: Comparing undesirable behaviours between ‘designer’ Poodle-cross dogs and their purebred progenitor breeds
Source: PLoS One. 2026 Mar 19;21(3):e0342847. doi: 10.1371/journal.pone.0342847 (PMC13001074; doi:10.1371/journal.pone.0342847)
Supplement: S4 File — (DOCX) [file pone.0342847.s004.docx]

| **Demographics** | **Categories** | **Designer-crossbreed** | **Purebreds** |
| --- | --- | --- | --- |
| Owner gender | Male | 341(10.0%) | 671 (11.2%) |
|  | Female | 2799 (81.7%) | 4693 (78.5%) |
|  | Prefer not to say | 7 (0.2%) | 19 (0.3%) |
| Owner age | 18 to 24 years old | 123 (3.6%) | 242 (4.0%) |
|  | 25 to 34 years old | 608 (17.8%) | 1050 (17.6%) |
|  | 35 to 44 years old | 649 (19.0%) | 973 (16.3%) |
|  | 45 to 54 years old | 779 (22.8%) | 1191 (20.0%) |
|  | 55 to 64 years old | 588 (17.2%) | 1163 (19.5%) |
|  | 65 to 74 years old | 280 (8.2%) | 596 (10.0%) |
|  | 75 years old + | 49 (1.4%) | 96 (1.6%) |
| First-time dog owner | yes | 1566 (45.7%) | 1799 (30.1%) |
|  | no | 1545 (45.1%) | 3554 (59.5%) |
| Primary carer of dog | yes | 1846 (53.9%) | 3411 (57.1%) |
|  | no | 19 (0.6%) | 32 (0.5%) |
| Employed in canine/animal care sector | yes | 153 (4.5%) | 657 (11.0%) |
|  | no | 2997 (87.5%) | 4753 (79.5%) |
| Did owner grow up with dog in childhood home | yes | 1454 (42.5%) | 2700 (45.2%) |
|  | no | 1677 (49.0%) | 2669 (44.6%) |
