# Supplementary material for: Comparing undesirable behaviours between ‘designer’ Poodle-cross dogs and their purebred progenitor breeds
Source: PLoS One. 2026 Mar 19;21(3):e0342847. doi: 10.1371/journal.pone.0342847 (PMC13001074; doi:10.1371/journal.pone.0342847)
Supplement: S5 File — (DOCX) [file pone.0342847.s005.docx]

| **C-BARQ behaviour scale** | **Poodle** | | **Cocker Spaniel** | | | | | **Cockapoo** | | | | | | | |
| --- | --- | --- | --- | --- | --- | --- | --- | --- | --- | --- | --- | --- | --- | --- | --- |
|  | **Descriptive** | | **Descriptive** | | **Multivariable vs Poodle** | | | **Descriptive** | | **Multivariable vs Poodle** | | | **Multivariable vs Cocker Spaniel** | | |
|  | **Mean** | **Mean 95% CI** | **Mean** | **Mean 95% CI** | **B_0_** | **95% CI** | **P value** | **Mean** | **Mean 95% CI** | **B_0_** | **95% CI** | **P value** | **B_0_** | **95% CI** | **P value** |
| (1) Trainability | 2.79 | 2.71-2.86 | 2.85 | 2.79-2.92 | 0.07 | 0.02-0.12 | **0.004** | 2.77 | 2.70-2.84 | -0.02 | -0.06-0.03 | 0.558 | -0.08 | -0.12- -0.05 | **<0.001** |
| (2) Owner-directed aggression | 0.05 | 0.03-0.06 | 0.05 | 0.03-0.06 | 0.00 | -0.01-0.01 | 0.940 | 0.07 | 0.05-0.08 | 0.02 | 0.01-0.03 | **0.005** | 0.02 | 0.01-0.03 | **<0.001** |
| (3) Stranger directed aggression | 0.15 | 0.12-0.18 | 0.09 | 0.07-0.11 | -0.06 | -0.08- -0.04 | **<0.001** | 0.18 | 0.15-0.20 | 0.03 | 0.01-0.05 | **0.017** | 0.09 | 0.07-0.11 | **<0.001** |
| (4) Dog-directed aggression | 0.19 | 0.15-0.22 | 0.13 | 0.10-0.16 | -0.06 | -0.08- -0.03 | **<0.001** | 0.19 | 0.16-0.22 | 0.00 | -0.03-0.03 | 0.876 | 0.06 | 0.04-0.08 | **<0.001** |
| (5) Dog rivalry | 0.08 | -0.05-0.12 | 0.08 | 0.06-0.11 | 0.01 | -0.02-0.03 | 0.581 | 0.12 | 0.08-0.14 | 0.03 | 0.00-0.06 | **0.024** | 0.02 | 0.00-0.05 | **0.032** |
| (6) Stranger-directed fear | 0.23 | 0.19-0.28 | 0.18 | 0.14-0.22 | -0.05 | -0.09- -0.01 | **0.009** | 0.27 | 0.23-0.32 | 0.04 | -0.00-0.08 | 0.059 | 0.09 | 0.06-0.12 | **<0.001** |
| (7) Non-social fear | 0.33 | 0.30-0.37 | 0.29 | 0.26-0.32 | -0.04 | -0.07- -0.01 | **0.004** | 0.39 | 0.35-0.42 | 0.05 | 0.02-0.08 | **0.001** | 0.09 | 0.07-0.12 | **<0.001** |
| (8) Touch sensitivity | 0.21 | 0.17-0.24 | 0.21 | 0.19-0.24 | 0.01 | -0.02-0.04 | 0.649 | 0.22 | 0.18-0.25 | 0.01 | -0.02-0.04 | 0.635 | 0.00 | -0.02-0.02 | 0.959 |
| (9) Dog-directed fear | 0.35 | 0.32-0.39 | 0.34 | 0.31-0.37 | -0.01 | -0.05-0.02 | 0.388 | 0.39 | 0.35-0.42 | 0.03 | -0.00-0.07 | 0.064 | 0.05 | 0.02-0.07 | **<0.001** |
| (10) Separation related problems | 0.60 | 0.53-0.66 | 0.46 | 0.41-0.52 | -0.13 | -0.19- -0.07 | **<0.001** | 0.67 | 0.61-0.74 | 0.08 | 0.02-0.14 | **0.010** | 0.21 | 0.16-0.26 | **<0.001** |
| (11) Excitability | 0.99 | 0.95-1.04 | 1.03 | 0.99-1.07 | 0.04 | -0.00-0.07 | 0.057 | 1.08 | 1.04-1.12 | 0.09 | 0.05-0.13 | **<0.001** | 0.05 | 0.02-0.08 | **<0.001** |
| (12) Attachment/  Attention seeking | 1.86 | 1.73-1.99 | 1.97 | 1.86-2.08 | 0.10 | -0.00-0.21 | 0.057 | 1.91 | 1.79-2.02 | 0.05 | -0.07-0.16 | 0.418 | -0.06 | -0.15-  0.03 | 0.194 |

**Table 1. General linear model analysis (ANOVA) of 12 C-BARQ behaviours in Cockapoos compared to their progenitor breeds . Coloured cells denote the ranking of scores within the sample population (red is highest, yellow is middle and blue is lowest). Figures in bold indicate statistically significant differences.**

Additional covariates were assessed in the general linear model and significant associations with any of the C-BARQ subscales (in brackets) were as : age of dog; ((4)*, (11)*, (12)*), sex; ((1)**, (2)**, (4)*, (10)***, (11)*), neuter status, age of owner; ((1)***, (3)**, (4)***, (6)***, (8)***, (10)***, (11)*), owner gender, whether puppy had been seen with the mother on day of pick up; ((1)***, (2)**, (11)**), whether owner worked in the canine sector; ((1)**, (2)**, (3)***, (7)***, (8)**, (10)***, (11)*, (12)**), primary dog ownership; ((3)*, (11)***), first-time ownership; ((1)*, (2)**, (5)*, (6)*, (7)***, (8)***, (10)**), overall disorder prevalence; ((1)***,(2)***,(3)***, (5)***,(6)***,(7)***,(8)***,(9)***,(10)***,(11)***). [[*** p <0.001, **p<0.01 and *p<0.05]

**Table 2. General linear model analysis (ANOVA) of 12 C-BARQ behaviours in Labradoodles compared to their progenitor breeds . Coloured cells denote the ranking of scores within the sample population (red is highest, yellow is middle and blue is lowest). Figures in bold indicate statistically significant differences.**

| **C-BARQ behaviour scale** | **Poodle** | | **Labrador Retriever** | | | | | **Labradoodle** | | | | | | | | | |
| --- | --- | --- | --- | --- | --- | --- | --- | --- | --- | --- | --- | --- | --- | --- | --- | --- | --- |
|  | **Descriptive** | | **Descriptive** | | **Multivariable vs Poodle** | | | **Descriptive** | | **Multivariable vs Poodle** | | | | **Multivariable vs Labrador Retriever** | | |  |
|  | Mean | Mean 95% CI | **Mean** | **Mean 95% CI** | **B_0_** | **95% CI** | **P value** | **Mean** | **Mean 95% CI** | **B_0_** | **95% CI** | **P value** | **B_0_** | | **95% CI** | **P value** |  |
| (1) Trainability | 2.77 | 2.71-2.82 | 2.93 | 2.88 – 2.98 | 0.17 | 0.10-0.21 | **<0.001** | 2.80 | 2.73-2.87 | 0.03 | - 0.03-0.10 | 0.300 | -0.13 | | -0.19- -0.08 | **<0.001** |  |
| (2) Owner-directed aggression | 0.04 | 0.14-0.05 | 0.02 | 0.01 – 0.03 | -0.03 | -0.03- -0.02 | **<0.001** | 0.02 | 0.01-0.03 | -0.02 | -0.03- -0.01 | **<0.001** | 0.00 | | -0.01-0.01 | 0.763 |  |
| (3) Stranger-directed aggression | 0.17 | 0.14-0.20 | 0.14 | 0.11 – 0.16 | -0.04 | -0.06- -0.02 | **0.001** | 0.16 | 0.13-0.19 | -0.01 | -0.04-0.02 | 0.364 | 0.02 | | -0.00-0.05 | 0.086 |  |
| (4) Dog-directed aggression | 0.22 | 0.18-0.25 | 0.15 | 0.12 – 0.18 | -0.07 | -0.10- -0.04 | **<0.001** | 0.18 | 0.14-0.21 | -0.04 | -0.08- -0.01 | **0.025** | 0.03 | | -0.00-0.06 | 0.081 |  |
| (5) Dog rivalry | 0.11 | 0.08-0.13 | 0.04 | 0.02 – 0.06 | -0.06 | -0.08- -0.05 | **<0.001** | 0.07 | 0.04-0.10 | -0.03 | -0.06- -0.01 | **0.007** | 0.03 | | 0.01-0.05 | **0.009** |  |
| (6) Stranger-directed fear | 0.23 | 0.18-0.28 | 0.17 | 0.13 – 0.21 | -0.06 | -0.10- -0.01 | **0.003** | 0.18 | 0.12-0.23 | -0.05 | -0.10-0.01 | **0.032** | 0.00 | | -0.04-0.04 | 0.303 |  |
| (7) Non-social fear | 0.33 | 0.29-0.37 | 0.26 | 0.23 – 0.30 | -0.07 | -0.09- -0.04 | **<0.001** | 0.33 | 0.29-0.37 | 0.00 | -0.04-0.04 | 0.937 | 0.07 | | 0.03-0.10 | **<0.001** |  |
| (8) Touch sensitivity | 0.23 | 0.19-0.27 | 0.23 | 0.20 – 0.27 | 0.01 | -0.02-0.04 | 0.576 | 0.23 | 0.18-0.27 | 0.00 | -0.04-0.04 | 0.983 | -0.01 | | -0.05-0.03 | 0.652 |  |
| (9) Dog-directed fear | 0.35 | 0.31-0.39 | 0.28 | 0.25 – 0.31 | -0.07 | -0.10-0.04 | **<0.001** | 0.30 | 0.25-0.34 | -0.05 | -0.09-0.01 | **0.009** | 0.02 | | - 0.02-0.05 | 0.112 |  |
| (10) Separation related problems | 0.62 | 0.56-0.68 | 0.30 | 0.24 – 0.35 | -0.33 | -0.37- -0.28 | **<0.001** | 0.54 | 0.47-0.60 | -0.09 | -0.15- -0.02 | **0.008** | 0.24 | | 0.19-0.30 | **<0.001** |  |
| (11) Excitability | 0.99 | 0.94-1.03 | 0.89 | 0.85 – 0.92 | -0.10 | -0.14- -0.07 | **<0.001** | 0.99 | 0.94-1.04 | 0.00 | -0.05-0.05 | 0.923 | 0.11 | | 0.06-0.15 | **<0.001** |  |
| (12) Attachment/  attention seeking | 1.87 | 1.74-2.00 | 1.74 | 1.62 – 1.85 | -0.13 | -0.23-0.03 | **0.012** | 1.78 | 1.64-1.93 | -0.09 | -0.22-0.05 | 0.220 | 0.05 | | -0.07-0.17 | 0.456 |  |

Additional covariates were assessed in the general linear model and significant associations with any of the C-BARQ subscales (in brackets) were as follows: age of dog; (6)**, sex; ((1)***, (2)*, (4)***, (9)*, (10)**, (11)*), neuter status; ((1)**, (2)*, (6)*, (7)*), age of owner; ((1)***, (4)*, (6)**, (8)*, (10)**), owner gender; (10)*, whether puppy had been seen with the mother on day of pick up; ((1)**, (10)**), whether owner worked in the canine sector; ((1)**, (5)*, (7)***, (8)*, (11)*), primary dog ownership; ((1)***,(4)**) and first-time ownership; ((1)***, (2)**, (7)***, (8)***, (10)**), overall disorder prevalence; ((1)***,(2)***,(3)***,(5)*,(7)***,(8)***,(9)**,(10)***,(11)***,(12)*). [*** p <0.001, **p<0.01 and *p<0.05]

**Table 3. General linear model analysis (ANOVA) of 12 C-BARQ behaviours in Cavapoos compared to their progenitor breeds. Coloured cells denote the ranking of scores within the sample population (red is highest, yellow is middle and blue is lowest). Figures in bold indicate statistically significant differences.**

Additional covariates were assessed in the general linear model and significant associations with any of the C-BARQ subscales (in brackets) were as follows: age of dog; (6)*, sex; ((1)*, (4)**, (10)*), neuter status; (2)**, age of owner; ((1)***, (3)*, (4)**, (6)**, (10)**, (11)***), owner gender; ((5)***, (9)*), whether puppy had been seen with the mother on day of pick up; ((1)***, (5)*), whether owner worked in the canine sector; ((1)*, (2)**, (3)*, (7)***, (8)*), primary dog ownership; ((1)*, (3)***) and first-time ownership; ((1)*, (2)*, (5)*, (7)***, (8)**, (10)**), overall disorder prevalence; ((1)***,(2)***,(3)***,(4)*,(5)***,(7)***,(8)***,(10)***,(11)*). [*** p <0.001, **p<0.01 and *p<0.05]

| **C-BARQ behaviour scale** | **Poodle** | | **CKCS** | | | | | **Cavapoo** | | | | | | | | |
| --- | --- | --- | --- | --- | --- | --- | --- | --- | --- | --- | --- | --- | --- | --- | --- | --- |
|  | **Descriptive** | | **Descriptive** | | **Multivariable vs Poodle** | | | **Descriptive** | | **Multivariable vs Poodle** | | | **Multivariable vs CKCS** | | | |
|  | **Mean** | **Mean 95% CI** | **Mean** | **Mean 95% CI** | **B_0_** | **95% CI** | **P value** | **Mean** | **Mean 95% CI** | **B_0_** | **95% CI** | **P value** | **B_0_** | **95% CI** | **P value** |  |
| (1) Trainability | 2.78 | 2.70-2.85 | 2.53 | 2.44-2.61 | -0.25 | -0.31- -0.19 | **<0.001** | 2.69 | 2.61-2.77 | -0.09 | -0.15 - -0.02 | **0.006** | 0.17 | 0.10-0.23 | **<0.001** |  |
| (2) Owner-directed aggression | 0.03 | 0.01-0.05 | 0.01 | -0.01-0.03 | -0.02 | -0.03- -0.00 | **0.029** | 0.04 | 0.02-0.05 | 0.01 | -0.01-0.02 | 0.379 | 0.02 | 0.01-0.04 | **0.003** |  |
| (3) Stranger-directed aggression | 0.16 | 0.13-0.19 | 0.05 | 0.02-0.09 | -0.10 | -0.13- -0.08 | **<0.001** | 0.15 | 0.12-0.18 | -0.01 | -0.03-0.02 | 0.534 | 0.10 | 0.07-0.12 | **<0.001** |  |
| (4) Dog-directed aggression | 0.19 | 0.14-0.23 | 0.09 | 0.05-0.14 | -0.09 | -0.13- -0.06 | **<0.001** | 0.16 | 0.12-0.20 | -0.03 | -0.06-0.01 | 0.123 | 0.07 | 0.03-0.10 | **<0.001** |  |
| (5) Dog rivalry | 0.10 | 0.06-0.13 | 0.08 | 0.04-0.12 | -0.02 | -0.05-0.01 | 0.281 | 0.11 | 0.07-0.15 | 0.01 | -0.02-0.04 | 0.455 | 0.03 | -0.00-0.06 | 0.085 |  |
| (6) Stranger-directed fear | 0.24 | 0.18-0.30 | 0.16 | 0.10-0.23 | -0.08 | -0.13- -0.03 | **0.003** | 0.25 | 0.18-0.31 | 0.01 | -0.04-0.06 | 0.758 | 0.09 | 0.03-0.14 | **0.002** |  |
| (7) Non-social fear | 0.34 | 0.29-0.39 | 0.28 | 0.23- -0.34 | -0.06 | -0.10- -0.02 | **0.007** | 0.38 | 0.33-0.43 | 0.04 | 0.01-0.08 | **0.027** | 0.10 | 0.06-0.14 | **<0.001** |  |
| (8) Touch sensitivity | 0.18 | 0.13-0.22 | 0.15 | 0.10-0.19 | -0.03 | -0.07-0.01 | 0.102 | 0.18 | 0.13-0.22 | -0.00 | -0.03-0.03 | 0.969 | 0.03 | -0.01-0.07 | 0.118 |  |
| (9) Dog-directed fear | 0.40 | 0.34-0.45 | 0.42 | 0.36-0.48 | 0.03 | -0.02-0.07 | 0.233 | 0.49 | 0.44-0.55 | 0.10 | 0.05-0.14 | **<0.001** | 0.07 | 0.02-0.12 | **0.003** |  |
| (10) Separation related problems | 0.60 | 0.51-0.69 | 0.51 | 0.41-0.61 | -0.09 | -0.16- -0.01 | **0.025** | 0.66 | 0.57-0.75 | 0.07 | -0.01-0.14 | 0.070 | 0.15 | 0.08-0.23 | **<0.001** |  |
| (11) Excitability | 0.99 | 0.94-1.05 | 0.94 | 0.88-1.00 | -0.05 | -0.10- -0.01 | 0.028 | 1.03 | 0.94-1.21 | 0.03 | -0.01-0.08 | 0.144 | 0.09 | 0.04-0.13 | **<0.001** |  |
| (12) Attachment/  Attention seeking | 1.89 | 1.73-2.06 | 1.84 | 1.66-2.02 | -0.05 | -0.19-0.08 | 0.445 | 1.83 | 1.66-2.00 | -0.07 | -0.20-0.07 | 0.325 | -0.01 | -0.15-0.13 | 0.860 |  |
